# Nlrc4 Inflammasome Expression After Acute Myocardial Infarction in Rats

**DOI:** 10.3390/ijms26083697

**Published:** 2025-04-14

**Authors:** Patricia Aparecida Borim, Mariana Gatto, Gustavo Augusto Ferreira Mota, Ana Luiza Barioni Meirelles, Anna Clara Consorti dos Santos, Luana Urbano Pagan, Elida Paula Benquique Ojopi, Eder Anderson Rodrigues, Lidiane Moreira Souza, Felipe Cesar Damatto, Leiliane Rodrigues dos Santos Oliveira, Leonardo Antonio Mamede Zornoff, Katashi Okoshi, Marina Politi Okoshi

**Affiliations:** 1 Internal Medicine Department, Botucatu Medical School, Sao Paulo State University (UNESP), Botucatu 18618-687, SP, Brazil; patricia.borim@unesp.br (P.A.B.); mariana.gatto@unesp.br (M.G.); gustavo.mota@unesp.br (G.A.F.M.); ana.meirelles@unesp.br (A.L.B.M.); anna.consorti@unesp.br (A.C.C.d.S.); luana.pagan@unesp.br (L.U.P.); eder.rodrigues@unesp.br (E.A.R.); lidiane.souza@unesp.br (L.M.S.); felipe.damatto@unesp.br (F.C.D.); lrs.oliveira@unesp.br (L.R.d.S.O.); leonardo.zornoff@unesp.br (L.A.M.Z.); katashi.okoshi@unesp.br (K.O.); 2 Clinic Hospital, Botucatu Medical School, Sao Paulo State University (UNESP), Botucatu 18618-687, SP, Brazil; benquique.ojopi@unesp.br

**Keywords:** myocardial ischemia, Nlrp3 Nlrp1, Nlrp1 inflammation, IL-1β

## Abstract

Acute myocardial necrosis activates the immune response and inflammatory processes. Although the initial response is helpful in restoring tissue injury, dysregulated and exacerbated inflammation contributes to the progression of cardiac remodeling. Inflammasomes play important roles in post-infarction inflammation. NALP1/NLRP1, NLRP 3, and NLRC4 are the best-known inflammasomes. NLRP3, which has received the most study in cardiovascular disease, has been linked to increased IL-1β (IL1B) production and caspase-1 activity, as well as impaired cardiac function. The role of NLRP1 and NLRC4 inflammasomes after acute myocardial infarction (MI) is poorly understood. We evaluated the expression of myocardial inflammasomes and inflammatory markers 72 h after MI in rats. Male Wistar rats were divided into Sham (n = 15) and MI (n = 16) groups. MI was induced by ligating the left anterior descending coronary artery. Infarct size was assessed by histology. Myocardial protein and gene expression was analyzed by Western blot and RT-qPCR, respectively. IL-1β (Il1b) concentrations in serum and heart macerate supernatant were evaluated by ELISA. Statistical analysis was performed using Student’s *t* test. Rats with an MI size less than 30% of the total left ventricle (LV) area were excluded; infarct size was 46 ± 11% of the total LV area in MI. The interstitial collagen fraction was higher in MI. Nlrc4, caspase-1 (Casp1), and IL-1β (Il1b) protein expressions were higher in MI. Nlrp3, Nlrp1, ASC (Pycard), pro-caspase-1, and pro-IL-1β (Il1b) expressions did not differ between groups. Expression of the Nlrp3 and ASC (Pycard) genes, as well as myocardial and serum IL-1β (Il1b) concentrations, was higher in MI. Acute post-myocardial infarction inflammation is characterized by increased protein expression of Nlrc4, caspase-1, and interleukin-1β; increased gene expression of Nlrp3 and ASC (Pycard); and elevated serum and myocardial concentrations of interleukin-1β in combination with an increased myocardial collagen interstitial fraction.

## 1. Introduction

Despite major advances in myocardial infarction (MI) diagnosis and treatment, morbidity and mortality rates are still high [1]. Acute myocardial ischemia leads to cardiomyocyte necrosis, which activates myocardial and systemic innate immune responses and an extensive inflammatory process [2]. The initial inflammatory response is helpful in restoring tissue injury; however, dysregulated inflammation exacerbates cardiac remodeling and heart failure [3,4,5].

Inflammasomes play important roles in post-infarction myocardial inflammation. Necrotic myocardial cells release molecules that act as danger signals, called damage-associated molecular patterns (DAMPs), and are recognized by different cell receptors expressed by immune cells, cardiomyocytes, and fibroblasts [6]. Some receptors, such as NOD-like receptors (NLRs), trigger the formation of inflammasomes, a multiprotein complex that releases and activates interleukin (IL)-1β and IL-18 (IL18). The NLR family pyrin domain-containing protein 1 (NALP/NLRP1) and protein 3 (NLRP3), as well as NLR family CARD domain-containing protein 4 (NLRC4) inflammasomes, are the best-known inflammasomes in immune and infectious diseases [7,8,9].

NLRP3 has undergone more study than other inflammasomes in cardiovascular disease. The activation of NLRP3 in post-MI fibroblasts contributes to an increase in IL-1β production [10]. Increased NLRP3 expression and caspase-1 activity have been observed after ischemic injury, and when inhibited, they reduce the infarcted area and attenuate cardiac dysfunction following ischemia/reperfusion in rodents [3,11]. Finally, NLRP3 activation after MI contributes to adverse cardiac remodeling and heart failure [12,13,14].

Despite extensive studies on NLRP3, the role of NLRP1 and NLRC4 in sterile inflammation, especially after MI, is poorly understood. NLRP1 and NLRC4 have classically been associated with inflammatory and infectious diseases [15,16,17]. Both inflammasomes are activated in the heart and endothelial cells of patients with chronic coronary disease [18]. NLRC4 was activated in the hearts of chronic cardiac failure patients, while NLRP1 remained unchanged [19]. Hyper NLRC4 and caspase-1 activation increased cardiomyocyte death, worsened cardiac function, and aggravated post-MI heart failure in experimental diabetes mellitus [20].

Very few studies have evaluated the participation of NLRP1 and NLRC4 inflammasomes in acute inflammation after ischemic damage [21]. The purpose of this study was to evaluate the expression of the myocardial NLRP1, NLRP3, and NLRC4 inflammasomes 72 h after MI in rats. We additionally evaluated the expression of important inflammatory markers such as caspase-1, ASC, and IL-1β.

## 2. Results

### 2.1. Experimental Groups

As perioperative mortality after MI induction is approximately 50% [22], we subjected 35 rats to surgery to induce MI and 17 to placebo surgery (Sham group). Nineteen rats from MI and two from Sham died during surgery and the perioperative period.

Anatomical variables are shown in Table 1. Atria weight values, atria-to-body weight (BW) ratio, and LV/BW ratio were higher in MI than Sham.

### 2.2. Histological Study

**Figure 1:**
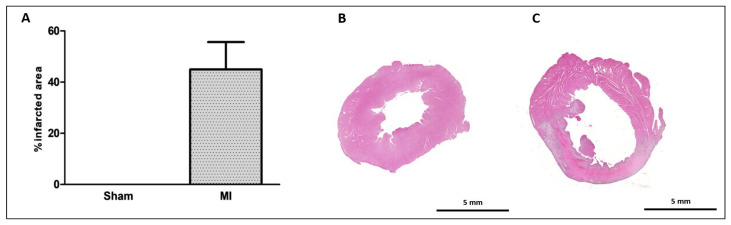
Myocardial infarction size. Infarcted area as a percentage of total left ventricle area (**A**); representative eosin–hematoxylin-stained left ventricle sections from Sham (**B**) and myocardial infarction (MI; (**C**)) groups. Data expressed as mean ± standard deviation. Sample size: Sham 9; MI 10.

**Figure 2:**
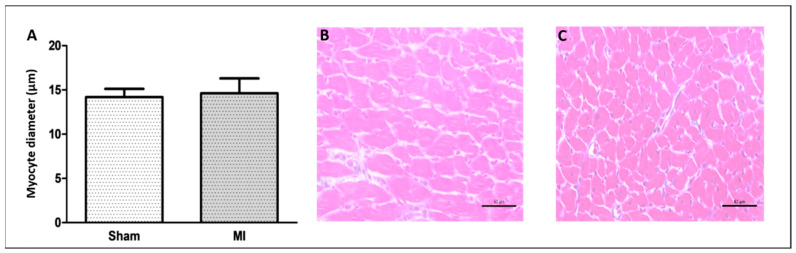
Left ventricle smallest myocyte diameter (**A**); representative eosin–hematoxylin-stained left ventricle sections from Sham (**B**) and non-infarcted area from myocardial infarction (MI; (**C**)) groups. Data are expressed as mean ± standard deviation. Bar scale: 50 µm; sample size: Sham 9; MI 11; Student’s *t* test.

**Figure 3:**
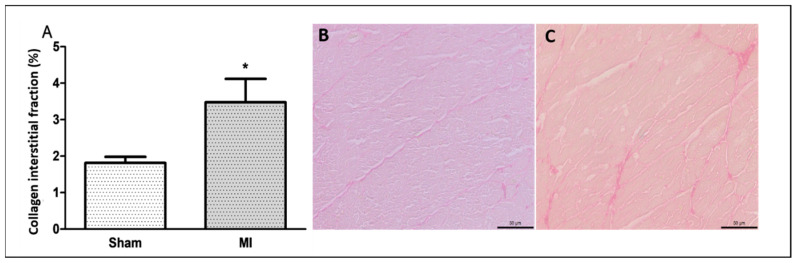
Percentage of left ventricle collagen interstitial fraction (**A**); representative *Picrosirius red*-stained left ventricle sections from Sham (**B**) and myocardial infarction (MI; (**C**)) groups. Data expressed as mean ± standard deviation. Bar scale: 50 µm; Student’s *t* test; * *p* < 0.001. Sample size: 10 per group.

The infarct size was 46 ± 11% of the total LV area in MI (Figure 1). Cardiomyocyte diameter did not differ between groups (Figure 2), and the interstitial collagen fraction was higher in MI (Figure 3).

### 2.3. Protein Expression

**Figure 4:**
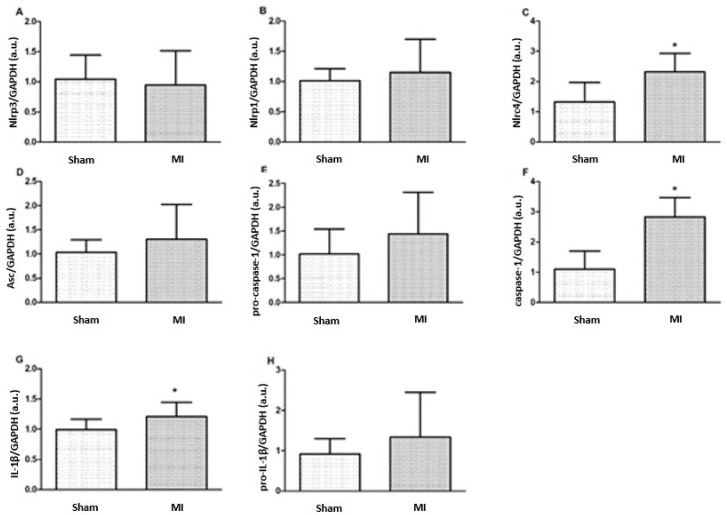
Protein expression of myocardial inflammasome components analyzed by Western blot: Nlrp3 (**A**), Nlrp1 (**B**), Nlrc4 (**C**), Asc (**D**), pro-caspase-1 (**E**), Caspase-1 (**F**), interleukin (IL)-1β (**G**), and pro-IL-1β (**H**). MI: myocardial infarction. Data expressed as mean ± standard deviation; Student’s *t* test; * *p* < 0.05. Sample size: Sham 15; MI 16.

**Figure 5:**
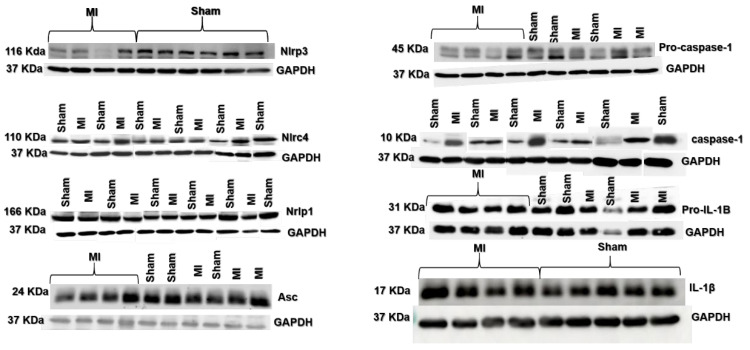
Representative blots of myocardial inflammasome components analyzed by Western blot.

Nlrc4, caspase-1, and IL-1β protein expressions were higher in MI (Figure 4 and Figure 5). Nlrp3, Nlrp1, Asc, pro-caspase-1, and pro-IL-1β expressions did not differ between groups.

### 2.4. IL-1β Concentration

**Figure 6:**
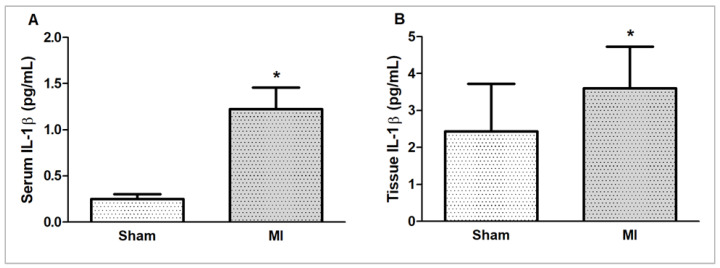
Serum (**A**) and myocardial (**B**) interleukin (IL)-1β concentrations evaluated by ELISA. MI: myocardial infarction. Sample size – serum (IL)-1β concentration: Sham 5; MI 6; myocardial IL-1β concentration: Sham 15; MI 16. Data expressed as mean ± standard deviation; Student’s *t* test; * *p* < 0.05.

Myocardial and serum concentrations of IL-1β were higher in MI (Figure 6).

### 2.5. Gene Expression

**Figure 7:**
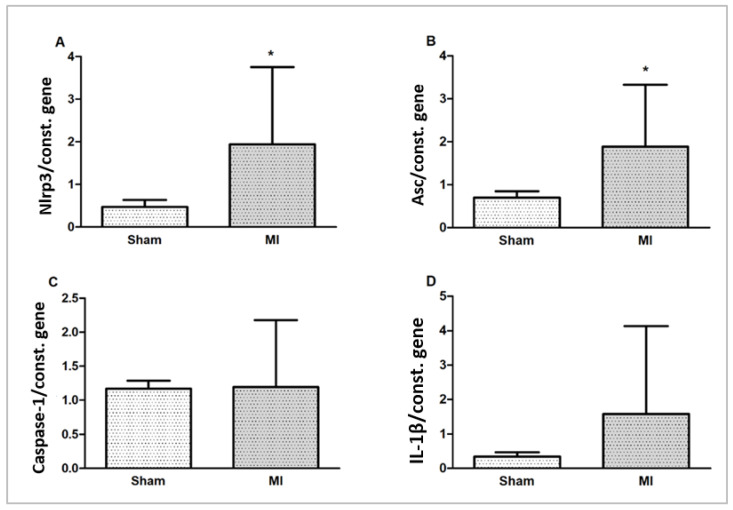
Myocardial gene expression of inflammasome components Nlrp3 (**A**), Asc (**B**), Caspase-1 (**C**), and interleukin (IL)-1β (**D**) normalized by the expression of the constitutive genes Gapdh and cyclophilin. Data expressed as mean ± standard deviation; Student’s *t* test; * *p* < 0.005.

Nlrp3 and Asc (Pycard) gene expressions were higher in MI. Caspase-1 and IL-1β gene expressions did not differ between groups (Figure 7).

## 3. Discussion

In this study, by evaluating the myocardial expression of inflammasomes 72 h after MI, a period characterized by acute inflammatory activation, we showed for the first time that NLRC4 is acutely activated after MI.

Despite the high perioperative mortality, the MI model in rats is extensively used to evaluate myocardial ischemia pathophysiology and treatment, because it is practical and low-cost compared to other animal models and demonstrates good result reproducibility in clinical studies. We have previously observed that the minimum infarct size to chronically cause functional and clinical abnormalities in rats is 38% and 40% of the total LV area, respectively [23]. In this study, our MI group had higher atria and LV weights and unchanged myocyte diameters. This is probably due to the acute LV and left atrium dilation caused by the large infarction size, which was 46 ± 11% of the total LV area. Interstitial edema may have also contributed to the higher weights of the left chambers.

Activation of myocardial inflammasomes is a complex event. Inflammasome sensors are usually characterized by a central nucleotide-binding and oligomerization domain (NACHT) and a C-terminal leucine-rich repeat domain (LRR). NLRP3 contains an amino-terminal pyrin domain (PYD); to recruit pro-caspase-1, it uses the adapter apoptosis-associated speck-like protein (ASC), which contains both the PYD and caspase-recruiting domain (CARD) and acts as a bridge between the sensor and pro-caspase-1. NLRP1 and NLRC4 sensors have a CARD in their N-terminal portion and can directly recruit pro-caspase-1 by CARD-CARD interaction [8,24]. NLRP1 and NLRC4 can also connect to ASC, leading to more efficient caspase-1 activation [25]. Sensor proteins can be sensitized by intra- and extracellular stimuli; after activation, they recruit protease caspase-1, which cleaves inactive precursors of IL-1β and IL-18 into their mature and active forms [26].

Studies have shown that the NLRP3, a key player in the inflammatory response, exhibits peak activation within the first days post-MI [27]. Therefore, we assessed myocardial inflammatory markers 72 h post-infarction. As in other studies [28,29,30], we observed higher protein caspase-1 expression in MI. Caspase-1 cleaves gasdermin D, which binds to plasma membrane lipids, forming pores and facilitating IL-1β release to the extracellular environment [28,31]. The plasma membrane pores cause electrolyte imbalance, water entry in cells, and myocyte rupture, a type of cell death called pyroptosis [28,31]. The contribution from caspase-1 to exacerbate inflammation and cardiac dysfunction is exemplified by the fact that genetic deletion of caspase-1 preserves cardiac function and reduces mortality, infarct size, and IL-1β in infarcted rodents and during ischemia/reperfusion injury [11,29,30].

Myocardial Nlrp3 and Asc gene expressions were higher in MI. However, the increased mRNAs did not translate into increased proteins levels. NLRP3 is a key mediator of the post-MI inflammatory response [10,11,13,31,32,33,34] and is involved in the development of atherosclerosis, MI, and heart failure [9]. NLRP3 can also mediate cell pyroptosis [9,35]. Although the persistence of increased NLRP3 levels was associated with continued inflammation and further cardiac dysfunction, the beneficial or harmful role of NLRP3 on injured cardiomyocytes has not been established [9].

ASC links the NLRP3 sensor to pro-caspase-1, which, by self-cleavage, becomes active caspase-1, forming a structure where pro-IL-1β is cleaved into the active form [36]. ASC is necessary for caspase-1 activation in the NLRP3 inflammasome, but not for activation of NLRP1 and NLRC4 inflammasomes, which have a CARD domain and can bind directly to caspase-1 [8,24]. The higher Asc was combined with an increased myocardial collagen interstitial fraction in MI, suggesting that increased Asc is involved in early myocardial fibrosis. ASC plays a key role in inflammasome activation, fibroblast activation, and collagen deposition, and the pathogenesis of myocardial fibrosis involves inflammatory signaling and profibrotic mediators [37,38]. Therefore, it is possible that the early increase in the myocardial interstitial collagen fraction was influenced by the higher Asc expression. Elevated ASC is linked to an increased risk of heart failure [39], while its gene deletion is associated with a smaller infarction size, less myocardial fibrosis, and improved ventricular function in infarcted mice [11]. A reparative phase with inflammation resolution and fibroblast proliferation has been reported 4 to 14 days after MI in mice [21]. Our data therefore show that myocardial collagen accumulation starts as early as 3 days after acute MI.

Nlrc4 protein expression was higher in MI. Classically, the NLRC4 is involved in the immune response against pathogens and autoimmune diseases and is part of the inflammasome complex that directly activates caspase-1. This process is important for the activation of pro-inflammatory cytokines such as IL-1β and the amplification of the inflammatory response [15,16,40,41]. Beyond inflammasome activation, NLRC4 also interacts with pathways like cGAS-STING by promoting TBK1 polyubiquitination and potentially affecting post-MI healing and cardiac remodeling [42]. Studies on NLRC4 activation in cardiovascular diseases are still preliminary. NLRC4 activation has been documented in human chronic heart failure and in rodents and pigs using different chronic heart failure models [18,19]. The role of the NLRC4 inflammasome in acute inflammation after ischemic damage is still unclear. However, our study shows for the first time that Nlrc4 protein expression is increased 3 days after MI.

The human NLRP1 inflammasome requires ASC to bind to caspase-1, while murine Nalp1b can directly bind to caspase-1 [43]. In this study, Nlrp1 inflammasome expression did not differ between groups, suggesting that, at least after acute post-ischemia, Nlrp1 is not needed to induce the inflammatory process. Myocardial NLRP1 did not differ between failing hearts and healthy subjects [19].

Myocardial and serum IL-1β concentrations, as well as myocardial IL-1β protein expression, were higher in MI. IL-1β plays a crucial role in the inflammatory response by increasing adhesion molecules and chemokines, which increases immune cells, endothelial cells, and fibroblasts in damaged sites; enhancing the production of complement system proteins and C-reactive protein; and boosting the phagocytic capacity of neutrophils and macrophages [44]. IL-1β also contributes to the perpetuation of inflammation [26]. Under physiological conditions, a small quantity of IL-1β is stored in its inactive form, which requires caspase-1 for cleavage and activation [6]. During acute inflammation, IL-1β is mainly produced by fibroblasts and immune cells; despite the presence of active caspase-1, cardiomyocytes produce small amounts of IL-1β [3,10,11]. Activation of inflammasomes in myocytes induces IL-1β production by other cell types, which contributes to worsening cardiac function [3,45,46]. Other studies have also reported increased myocardial and serum IL-1β after acute infarction [11,47].

In summary, large-sized MI acutely increased the weights of the left chambers and myocardial fibrosis in rats. Post-infarction myocardial inflammation was characterized by increased Asc and Nlrp3 gene expression and caspase-1, IL-1β, and Nlrc4 protein expression. There has been extensive investigation on the role of the NLRP3 inflammasome in the inflammatory process after acute and chronic MI. However, this study highlights that the Nlrc4 inflammasome is also acutely activated after MI, and, therefore, research is needed to establish its role in post-MI chronic cardiac remodeling. Understanding the participation of different inflammasomes after infarction should contribute to the development of drugs aimed at preserving ventricular function and attenuating heart failure in infarction patients.

## 4. Materials and Methods

### 4.1. Experimental Animals and Study Protocol

Male 200 to 250 g Wistar rats were purchased from the Central Animal Center, Botucatu Medical School, Sao Paulo State University, UNESP, Brazil. Animals received food and water ad libitum and were kept in collective cages with three rats per cage in a temperature-controlled environment (24 ± 2 °C) with 12 h light/dark cycles. This study was approved by Botucatu Medical School Animal Research Ethics Committee, Sao Paulo State University, UNESP, Botucatu, SP, Brazil (protocol nº 1307/2019). All experiments were conducted in accordance with the Guide for the Care and Use of Laboratory Animals published by the National Research Council, 2011.

Rats were randomly assigned to Sham or MI groups. MI was induced by ligation of the left anterior descending coronary artery, as previously described [48,49]. Control animals underwent the same surgical procedure without coronary artery ligation.

### 4.2. Tissue Collection

Seventy-two hours after MI induction, the rats were weighed, anesthetized with intraperitoneal sodium thiopental (120 mg/kg), and euthanized. After blood was collected, hearts were removed by thoracotomy. Atria and ventricles were dissected and weighed. The left ventricle (LV) and serum samples were frozen in liquid nitrogen and stored at −80 °C.

### 4.3. Infarction Size

The degree of heart injury from MI was determined by assessing infarcted area percentage in relation to total LV area. An LV slice was cut 5 to 6 mm from the apex, reflecting the average result from the entire ventricle [50]. Samples were fixed in 10% buffered formalin solution for 24 h, washed in water, and transferred to 70% ethanol. LV sections were stained with hematoxylin–eosin and examined by a microscope (Leica DM LS, Nussloch, BW, Germany) coupled to a computerized imaging analysis system (Media Cybernetics, Silver Spring, MD, USA). Infarction size was calculated by dividing the sum of endocardial and epicardial infarcted ventricular lengths by the sum of the total (infarcted and viable myocardium) endocardial and epicardial ventricular circumferences [51]. Values were expressed as a percentage of the total LV area. As infarction size is highly variable after left anterior descending coronary artery ligation in rats, only animals with an MI > 30% of the total LV area on histological evaluation were included in the study.

### 4.4. Histological Analysis

Serial transverse 8 μm thick sections were cut from the non-infarcted LV in a cryostat cooled to −20 °C and stained with hematoxylin and eosin. The smallest diameters passing through the nucleus of 150 to 200 cardiomyocytes were measured [52]. Other slides were stained with Picrosirius red and used to quantify interstitial collagen fraction. On average, 20 microscopic fields were analyzed with a 40× lens. Perivascular collagen was excluded from the analysis [53].

### 4.5. Western Blot

Myocardial tissue was homogenized in RIPA buffer containing protease (Sigma-Aldrich, St. Louis, MO, USA) and phosphatase (Roche Diagnostics, Indianapolis, IN, USA) inhibitors using a bead beater homogenizer (Bullet Blender^®^, Next Advance, Inc., Troy, NY, USA) and centrifuged at 12,000× *g*, at 4 °C, for 20 min [54,55,56]. Protein concentrations were assessed using the Pierce BCA Protein Assay Kit (Thermo Fisher Scientific, Waltham, MA, USA). A total of 40–50 μg of protein lysate was resolved by SDS-PAGE and transferred to a nitrocellulose membrane (Armsham Biosciences, Piscataway, NJ, USA). The blotted membrane was blocked with 5% non-fat dry milk in TBS-T (20 mmol/L Tris-HCl pH 7.4, 137 mmol/L NaCl and 0.1% Tween 20) for 1 h at room temperature and incubated overnight at 4–8 °C with the following primary antibodies: anti-Nlrp3 (1:500, Thermo Fisher Scientific, Waltham, MA, USA), Nlrc4 (1:500, Invitrogen, Waltham, MA, USA), Nlrp1, Asc, Casp1, and Il1b (1:200, Santa Cruz Biotechnology, Dallas, TX, USA). Blots were then incubated for 1.5 h at room temperature with a secondary antibody anti-rabbit IgG-HRP (Abcam, Cambridge, MA, USA) or anti-mouse IgG-HRP (Abcam) followed by chemiluminescence detection with the SuperSignal^®^ West Pico kit (Thermo Fisher Scientific, Waltham, MA, USA) using Image Quant™ 800 (IQ800, GE Healthcare, Waukesha, WI, USA). Quantification of band intensities was performed using Image-J software (version 1.53t, National Institutes of Health, Bethesda, MD, USA). The results were normalized to Gapdh (1:5000, Abcam, Cambridge, MA, USA) [57,58].

### 4.6. Cytokine Concentration

The concentration of IL-1β (Il1b) in heart homogenate and serum was quantified by ELISA. A DuoSet Systems Kit (R&D Systems, Minneapolis, MN, USA) was used according to the manufacturer’s instructions. Data acquisition and analysis were performed using Gen5 Microplate Reader and Image Software (version 3.12, BioTek Instruments, Winooski, VT, USA).

### 4.7. Gene Expression by qRT-PCR

Myocardial gene expression was evaluated by Real-Time Quantitative PCR (RT-q-PCR). Total RNA was extracted using TRIZOL^®^ (Ambicon Life Technologies, Carlsbad, CA, USA) according to the manufacturer’s instructions. RNA concentrations were quantified using a Nano Drop spectrophotometer (Thermo Fisher Scientific, Waltham, MA, USA) with absorbance values at 260 nm and expressed in ng/µL. All samples had an absorbance ratio of approximately 2.0 at 260/280 nm.

The cDNA was synthesized from 1 μg of total RNA using a High-Capacity cDNA Reverse Transcription Kit (Applied Biosystems, Foster City, CA, USA), following the manufacturer’s instructions. Nlrp3 (Rn04244620_m1), Casp1 (Rn00562724_m1), Asc (Rn00597229_g1), and Il1b (Rn01514151_m1) genes were amplified by qPCR in a StepOne™ Real-Time PCR System (Applied Biosystems) with the TaqMan™ Gene Expression Master Mix kit (Thermo Fisher Scientific). Cyclophilin (Rn00690933_m1) and Gapdh (Rn01775763_g1) were used as housekeeping controls to normalize the relative fluorescence signal of the target genes. Each sample was analyzed in duplicate. Relative expression (fold-change: 2^−∆∆ct^) of the interest genes was calculated by normalizing the threshold cycle (Ct) values to the reference genes [59,60,61].

### 4.8. Statistical Analysis

Results are presented as mean ± standard deviation or median and quartiles, according to the distribution. Comparisons between groups were performed by Student’s *t* test or the Mann–Whitney test. Analyses were performed using SigmaPlot V 12.0 software (Palo Alto, CA, USA). A 5% significance level was considered.

## 5. Conclusions

Myocardial infarction acutely induces inflammation, which is characterized by increased protein expression of NLRC4, caspase-1, and interleukin-1β; increased gene expression of NLRP3 and ASC; and increased serum and myocardial concentrations of interleukin-1β in combination with an increased myocardial collagen interstitial fraction.

## Figures and Tables

**Table 1:** Anatomical variables.

	Sham (n = 15)	MI (n = 16)
BW (g)	263 ± 31	249 ± 16
LV (g)	0.55 (0.49–0.65)	0.59 (0.57–0.64)
LV/BW (mg/g)	2.17 ± 0.15	2.44 ± 0.17 *
RV (g)	0.19 ± 0.03	0.20 ± 0.03
RV/BW (mg/g)	0.74 ± 0.09	0.80 ± 0.11
Atria (g)	0.07 ± 0.01	0.11 ± 0.03 *
Atria/BW (mg/g)	0.27 ± 0.05	0.45 ± 0.12 *

MI: myocardial infarction; n: sample size; BW: body weight, LV: left ventricle weight; RV: right ventricle weight. Data expressed as mean ± standard deviation or median and percentiles; Student’s *t* test or Mann–Whitney; * *p* < 0.001.

## Data Availability

Restrictions apply to the datasets.
